# Recent Advances in the Cellular and Developmental Biology of Phospholipases in Plants

**DOI:** 10.3389/fpls.2019.00362

**Published:** 2019-04-05

**Authors:** Tomáš Takáč, Dominik Novák, Jozef Šamaj

**Affiliations:** Department of Cell Biology, Centre of the Region Haná for Biotechnological and Agricultural Research, Faculty of Science, Palacký University Olomouc, Olomouc, Czechia

**Keywords:** cellular functions, phospholipases, phospholipase A, phospholipase C, phospholipase D, plant development, phosphatidic acid, phytohormones

## Abstract

Phospholipases (PLs) are lipid-hydrolyzing enzymes known to have diverse signaling roles during plant abiotic and biotic stress responses. They catalyze lipid remodeling, which is required to generate rapid responses of plants to environmental cues. Moreover, they produce second messenger molecules, such as phosphatidic acid (PA) and thus trigger or modulate signaling cascades that lead to changes in gene expression. The roles of phospholipases in plant abiotic and biotic stress responses have been intensively studied. Nevertheless, emerging evidence suggests that they also make significant contributions to plants’ cellular and developmental processes. In this mini review, we summarized recent advances in the study of the cellular and developmental roles of phospholipases in plants.

## Introduction

Phospholipids are major components of biological membranes and have important signaling functions (Hou et al., 2016). Phospholipases (PLs) represent a ubiquitous family of proteins that cleave various bonds in phospholipids to maintain membrane lipid homeostasis and stability (Wang et al., 2012). Plant PLs are divided into three major classes: A, C, and D. These classes are distinguished by the type of catalytic reactions they perform. Phospholipases A (PLAs) hydrolyze acyl groups from the *sn-1* and *sn-2* positions of phospholipids and produce free fatty acids and lysophospholipids. Phospholipases C (PLCs) catalyze the hydrolysis of the entire phosphoryl head group, cleaving the glycerophosphate bond and thus yielding diacylglycerol and the phosphorylated head group. Phospholipases D (PLDs) cleave the phosphodiester (phosphorus-oxygen) bond in the phospholipid, yielding phosphatidic acid (PA; Chen et al., 2011). The hydrolytic activity of PLs is often accompanied by the production of lipid second messengers, which mediate plant responses to external stimuli. Notably, PLs have the ability to bind and modulate other regulatory proteins, including the subunits of G-protein complexes and sphingosine kinase (Mishra et al., 2006; Guo et al., 2012; Pandey, 2016). Their role in membrane lipid remodeling and the subsequent regulation of membrane physical properties makes PLs and their hydrolytic products key regulators of endomembrane organization. In addition, PLs also control the organization and dynamics of the cytoskeleton (Zhang et al., 2012; Pleskot et al., 2013). They are involved in jasmonic acid (JA) biosynthesis (Ishiguro et al., 2001) and also have notable impact on plant responses to such hormones as abscisic acid (ABA; Mishra et al., 2006; Peters et al., 2010; Uraji et al., 2012), auxins (Scherer et al., 2012), and cytokinins (Repp et al., 2004). The interplay of PLs with hormones strongly regulates plant developmental processes, as indicated by the phenotypes of mutants with deficient PL expression (e.g., Labusch et al., 2013). Through all of these mechanisms, PLs are involved in plant responses to drought (Sang et al., 2001), chilling (Huo et al., 2016), heat (Krčková et al., 2015), salt (Arisz and Munnik, 2011), osmotic pressure (Hong et al., 2008), heavy metal stress (Pejchar et al., 2015), phosphorus deficiency (Cruz-Ramírez et al., 2006; Li et al., 2006b), and pathogens (Zhao, 2015). Last but not least, PLs modulate plant developmental processes, such as pollen tube growth (Potocký et al., 2003; Kim et al., 2011), embryogenesis, and root and leaf development (Wimalasekera et al., 2010), as well as gravitropism (Lee, 2003).

The heterogeneity of PLs indicates that they carry specific developmental functions with different degrees of redundancy within or across particular PL subfamilies. The involvement of PLs in the abiotic and biotic stress responses of plants has been frequently and comprehensively reviewed in recent studies (Wang, 2014; Hong et al., 2016), while their role in plant developmental processes is largely underestimated. This mini review aimed to summarize the recent advances in the study of the roles of PLs in plant developmental processes, which have been mostly related to their cellular functions.

## Cellular and Developmental Roles of Phospholipases A

PLAs are a multigene family of enzymes that are subdivided into PLA_1_s, secretory PLAs (PLA_2_s) and patatin-like PLAs (pPLAs). They show affinities for diverse phospholipid substrates, including phosphatidylcholine (PC), monogalactosyldiacylglycerol, digalactosyldiacylglycerol, and triacylglycerol (Chen et al., 2013).

**Figure 1:**
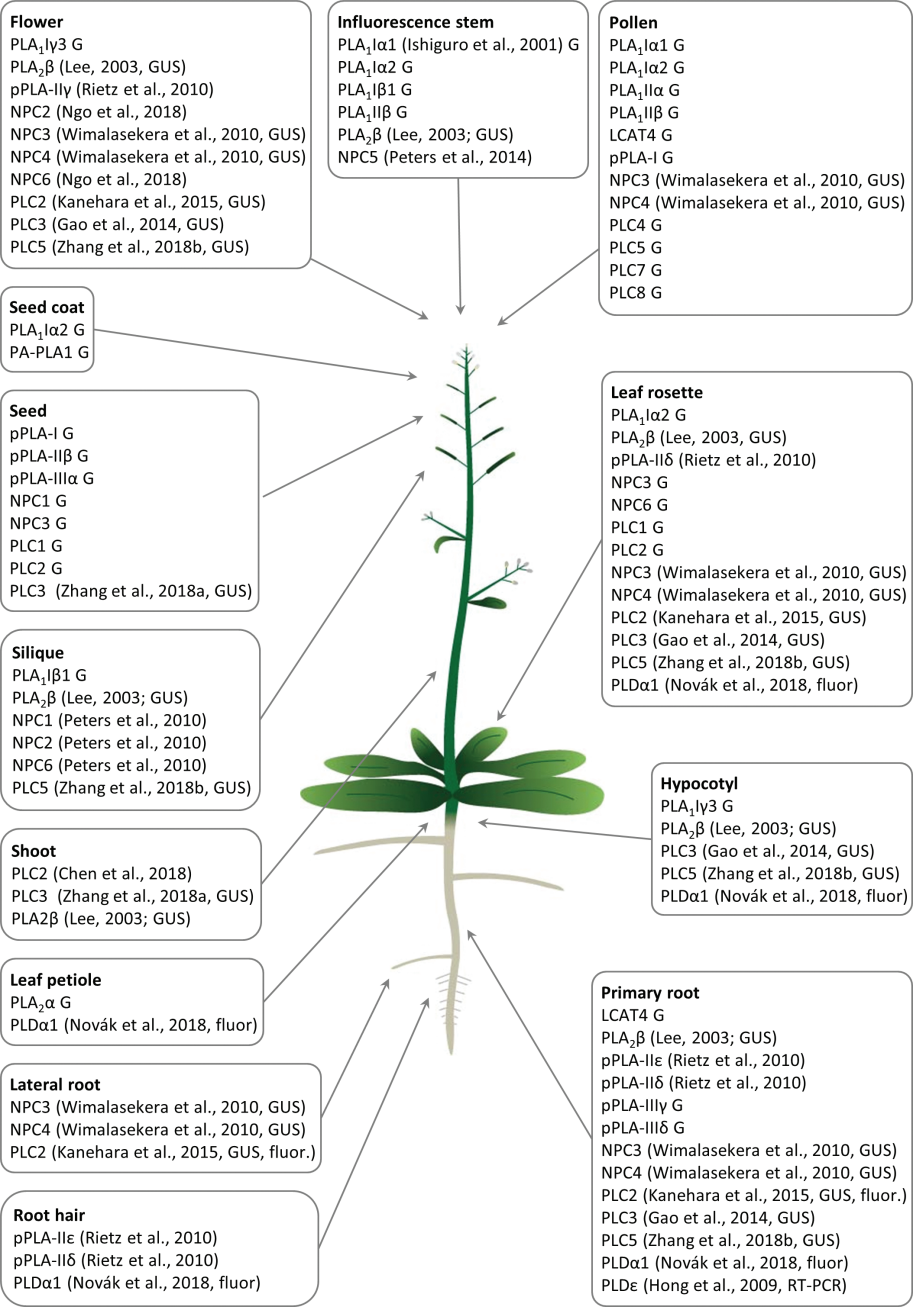
Overview of *Arabidopsis* tissue- and organ-specific expression patterns and enrichment of diverse phospholipases important for the development of respective plant organs and tissues. G means that the gene expression data were retrieved from the Genvestigator database, GUS means that the expression data represented the results of promoter-GUS reporter gene assays, and fluor means that the expression data represented microscopic observations of fluorescently tagged phospholipases. Citations to the relevant original articles are provided.

**Figure 2:**
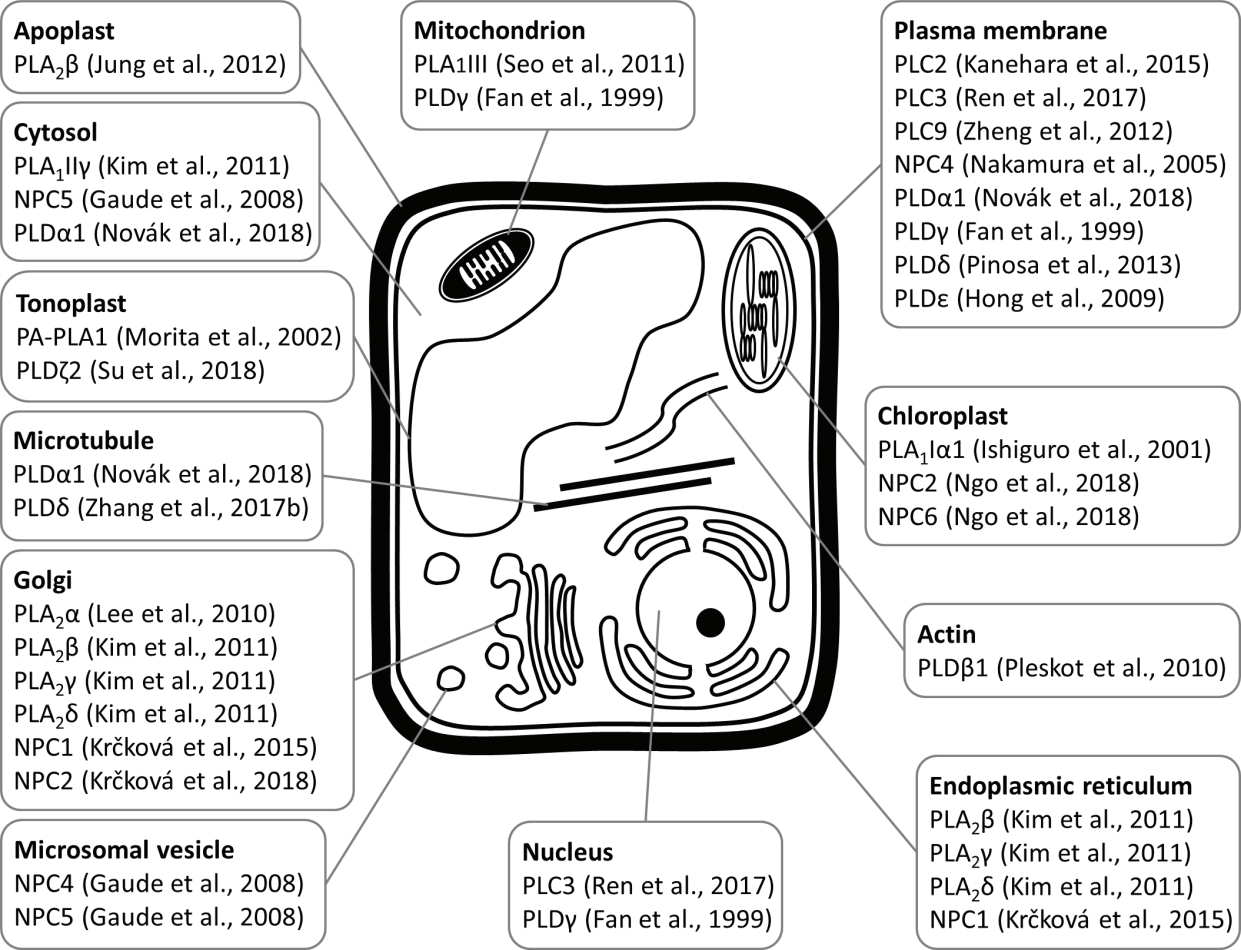
Schematic overview of subcellular localizations of plant phospholipases to diverse subcellular compartments and organelles, as revealed by experimental approaches with relevant citations to the original articles.

**Figure 3:**
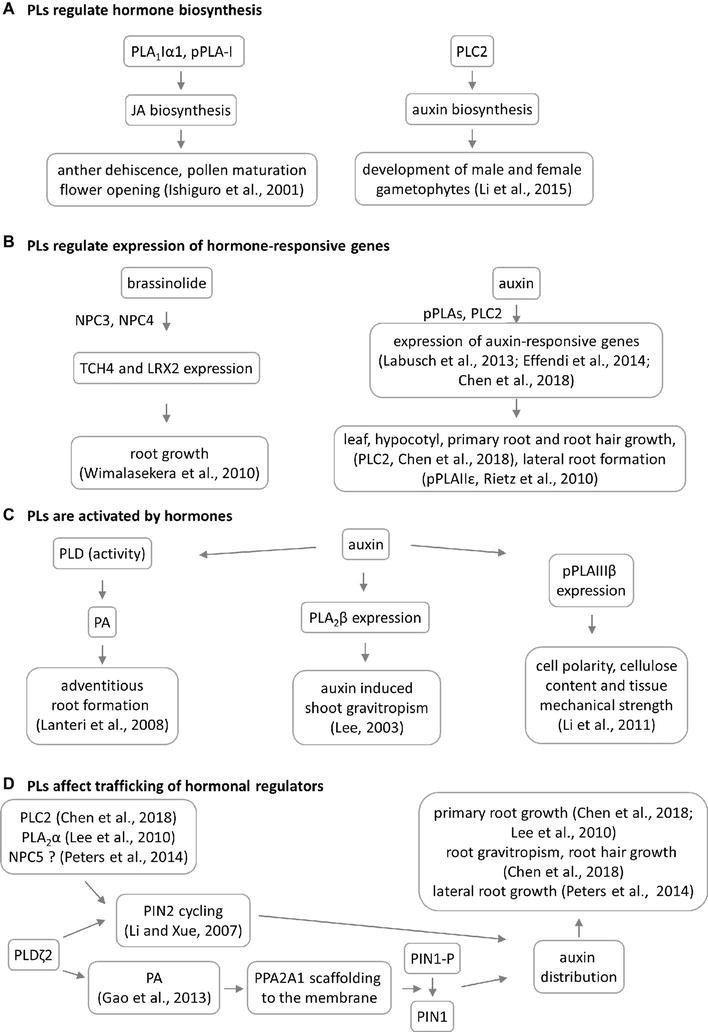
Overview of crosstalk between phospholipases (PLs) and hormones during plant development. Four models of PL–hormone interaction are proposed. **(A)** PLs are involved in jasmonic acid and auxin biosynthesis. This has consequences in reproductive organ development. **(B)** PLs are regulating the hormone-induced gene expression having impact on root and shoot development, TCH4 = xyloglucan endotransglycosylase 4, LRX2 = leucine-rich extensin 1. **(C)** PLs are activated by hormones leading to developmental phenotypes. In some cases, the PL mutation does not lead to phenotypic manifestation. **(D)** Particular PLs have impact on the auxin distribution through 2 different mechanisms. NPC5 promotes lateral root formation likely through regulating auxin transport and distribution (Peters et al., 2014), which occurs *via* PIN2 cycling.

PLA_1_ enzymes are calcium-independent and catalyze the hydrolysis of acyl groups from the *sn-1* position of phospholipids (Chen et al., 2013). Their developmental expression and localization is very elusive. PLA_1_Iα1 is targeted to the chloroplasts and is expressed specifically in the stamens before flower opening (Ishiguro et al., 2001). PLA_1_III is expressed at high levels in young seedlings and localized to the mitochondria (Figure 2; Seo et al., 2011). Some members of this subfamily are expressed at high levels in the pollen (PLA_1_Iα1, PLA_1_Iα2, PLA_1_IIα, and PLA_1_IIβ), petals and sepals (PLA_1_Iγ3), seed coats (PLA_1_Iα2), inflorescence stems (PLA_1_Iα1, PLA_1_Iα2, PLA_1_Iβ1, and PLA_1_IIβ), and xylem of the hypocotyl stele (PLA_1_Iγ3) in *Arabidopsis* (Figure 1). They show increased expression during young rosette development (PLA_1_Iα2), silique maturation, and senescence (PLA_1_Iβ1; Genvestigator). These findings indicate that these PLA_1_s have prominent roles in generative organ development (Figure 1). For example, processes such as anther dehiscence, pollen maturation, and flower opening are controlled by the chloroplast-associated PLA_1_Iα1 (Figure 2), which was proposed to prefer PC as a substrate (Ishiguro et al., 2001). It was shown experimentally that this regulation is mediated by the participation of PLA_1_Iα1 in chloroplast-dependent JA biosynthesis (Figure 3A). Notably, PLAs catalyze the release of α-linolenic acid, which is a precursor of JA, from membrane phospholipids (Wasternack and Song, 2016). This supports the role of PLAs in JA-controlled developmental processes. Remarkably, PLA_1_Iα1 is not only linked to JA, as its expression is also dependent on AUXIN RESPONSE FACTOR 6 and 8 (Tabata et al., 2010), ubiquitin ligase DAF (Peng et al., 2013), and the floral homeotic gene *AGAMOUS* (Ito et al., 2007). Nevertheless, PLA_1_ functions are not restricted only to generative organ development. The vacuolar membrane-localized PA-PLA1 (also known as SHOOT GRAVITROPISM 2; SGR2) is important in the regulation of shoot gravitropism. This role is linked to vacuole biogenesis, a process that is inevitably involved in amyloplast redistribution in the shoot endodermis (Kato et al., 2002; Morita et al., 2002). Recently, a similar function of PA-PLA1 was discovered in the zygote, where it controls the polar vacuole positioning to ensure asymmetrical cell division (Kimata et al., 2019).

The gene *FSE1* encodes a PL-like protein that is homologous to the PA-preferring PLA_1_s and controls the synthesis of galactolipids in the rice endosperm. It is important for seed and endosperm development (Long et al., 2018). Furthermore, PLA_1_s may also have root-specific functions. A pepper PLA_1_ is expressed selectively in young, growing roots and regulates growth rates and root and leaf tissue patterning. The authors of the previous study (Seo et al., 2008) that examined it proposed that this PLA_1_ may be involved in cell cycle control *via* the signaling functions of its hydrolytic products. Recently, a beneficial biotechnological application of this PLA_1_ was presented, as the yields of transgenic rice plants overexpressing pepper PLA_1_ were increased relative to normal plants (Park et al., 2016).

Similar to PLA_1_s, only limited information exists on the developmental expression of most PLA_2_s. These are also called secretory PLAs and catalyze phospholipid hydrolysis at the *sn-2* position. PLA_2_s are expressed at high levels in pollen (lecithin-cholesterol acyltransferase-like 4; LCAT4), leaf petioles (PLA_2_α), the root pericycle (LCAT4; Figure 1), and flowers (PLA_2_γ; Bahn et al., 2003). They produce lysophospholipids (Lee et al., 2005; Chen et al., 2013) capable of inducing local positive membrane curvature (Boutté and Moreau, 2014), which is important for endomembrane morphogenesis. PLA_2_α, PLA_2_β, PLA_2_γ, and PLA_2_δ are localized in the Golgi apparatus and endoplasmic reticulum (ER; Lee et al., 2010; Kim et al., 2011), while PLA_2_β is secreted into the apoplast (Bahn et al., 2003; Lee, 2003). Specifically, PLA_2_β is gradually translocated to the apoplast during leaf maturation (Figure 2; Jung et al., 2012).

PLA_2_β controls cell elongation in the leaf petioles and inflorescence stems (Lee, 2003). It is also implicated in shoot (but not root) gravitropism *via* an auxin-dependent mechanism, which is seemingly different from that of the abovementioned PA-PLA1 (Figure 3; Lee, 2003; Kimata et al., 2019). The interplay between PLA_2_s and auxin was previously explained mechanistically. It was found that PLA_2_α and the product of its lipid-hydrolyzing activity, lysophosphatidyl ethanolamine (LPE), are both required for the trafficking of PINs (auxin efflux transporters) to the plasma membrane (PM). This is required for proper root growth in *Arabidopsis* (Figure 3D; Lee et al., 2010). Interestingly, PLA_2_-mediated production of LPE is also important for the proper organization of the endomembrane system during pollen development, germination, and elongation (Kim et al., 2011). This finding enriched the list of important signaling lipids involved in determining polar tip growth.

Patatin-related PLAs (pPLAs) hydrolyze a broad range of phospholipids at both the *sn-1* and *sn-2* positions and contain a patatin-catalytic domain. In addition, they can also use galactolipids as substrates. The pPLAs in *Arabidopsis* are classified into three subfamilies (pPLA-I, pPLA-II, and pPLA-III) (Scherer et al., 2010; Li and Wang, 2014), although the individual members of these subfamilies might possess different substrate specificities (Rietz et al., 2010). *Arabidopsis* pPLA-IIε and pPLA-IIδ are expressed at high levels in the roots and root hairs, and pPLA-IIδ is expressed also in the leaves and cotyledons. Expression of pPLA-IIγ is restricted to the generative organs (Rietz et al., 2010). Other pPLAs are expressed at high levels in the pollen (pPLA-I), seeds (pPLA-I, pPLA-IIβ, and pPLA-IIIα), and roots (pPLA-IIIγ and pPLA-IIIδ; Figure 1). The overexpression of pPLA-IIIδ leads to stiff inflorescence stems, thicker leaves, shorter siliques, larger seeds, more rounded flowers, and delayed plant growth (Huang, 2001).

There is solid evidence of the occurrence of crosstalk between pPLAs and plant hormones. While pPLA-I is involved in jasmonic acid biosynthesis, *pPLA-IIδ* and *pPLAIIIβ* expression is strongly induced by auxin (Rietz et al., 2010; Li et al., 2011). Interestingly, it was also discovered that pPLAs are important for the expression of early auxin-responsive genes in *Arabidopsis* (Figure 3B; Labusch et al., 2013; Effendi et al., 2014). The knock-out mutants *pplaIIIβ* and *pplaIIε* do not exhibit auxin-related phenotypes under normal growth conditions (Rietz et al., 2010; Li et al., 2011), while the *pplaIIIδ* mutant consistently showed higher sensitivity to auxin (Labusch et al., 2013). Crosstalk between pPLAs and auxin likely affects cell polarity, cellulose content, and tissue mechanical strength in *Arabidopsis*, rice, and ginseng (Figure 3C; Li et al., 2011; Liu et al., 2015; Jang and Lee, 2019). Since cellulose deposition is tightly controlled by microtubules, it would be extremely challenging to further investigate the interplay between pPLAIIIβ-induced lipid remodeling, cellulose deposition, and microtubule dynamics. This assumption is also substantiated by the fact that pPLAIIIδ overexpression inhibited longitudinal growth but promoted transverse growth, in most organs of *Arabidopsis* and *Brassica napus* (Dong et al., 2014). Growth polarity appears to be under the control of auxin and ethylene, and these plant hormones are involved in growth reprogramming through modifications of microtubule orientation governed by katanin (Lindeboom et al., 2013; Sassi et al., 2014; Luptovčiak et al., 2017). Proof of this concept was provided by the differential proteomic analysis of pPLAIIIδ-overexpressing plants, which demonstrated the impact of this protein on the abundance of the microtubule-associated protein MAP18, which modulates directional cell growth (Zheng et al., 2014).

## Cellular and Developmental Roles of Phospholipases C

The phospholipid-hydrolyzing activity of PLCs on the phosphodiester bond results in the production of diacylglycerol (DAG) and head group (Singh et al., 2015; Hong et al., 2016). DAG may then be converted into PA by DAG kinase (Arisz et al., 2009), so the developmental impacts of PLCs might also be assigned to their product, PA. This was previously tested by a pharmacological approach employing PLC and PLD inhibitors (den Hartog et al., 2001). Nevertheless, PLC- and PLD-mediated PA production pathways may differ in their functions, as exemplified by their effects on pollen tube growth. Pharmacological inhibition of both PLC and PLD caused pollen tube growth to cease, while cellular processes essential for polarized growth exhibited different sensitivities to PLC and PLD inhibitors. Vacuole organization was heavily altered by PLC inhibitor, while actin dynamics was affected specifically by pharmacological inhibition of PLD (Pleskot et al., 2012). PLCs in plants can be divided into three groups according to their substrate specificity and cellular functions. Non-specific PLCs (NPCs; in *Arabidopsis* these were designated as NPC1-NPC6) act on common phospholipids, such as PC and PE (Pokotylo et al., 2013). Phosphoinositide-specific PLCs (PI-PLCs; in *Arabidopsis* designated as PLC1-PLC9) hydrolyze phosphoinositides, such as phosphatidylinositol 4,5-bisphosphate (PIP2) (Tasma et al., 2008; Heilmann, 2016). Glycosylphosphatidylinositol (GPI)-specific PLCs cleave GPI anchors from proteins (Hong et al., 2016). To our knowledge, no PL with GPI-PLC activity has been characterized in plants so far.

Previous studies reported that NPC1, NPC2, and NPC6 were expressed at high levels in siliques, while NPC3 and NPC4 were expressed at high levels in roots and inflorescences. NPC5 expression is similar to that of NPC3 and NPC4, although its expression is absent in roots (Peters et al., 2010, 2014; Wimalasekera et al., 2010). The expression of NPC2 and NPC6 is developmentally regulated, and at later stages, they show rather limited expression in vegetative tissues but pronounced expression in reproductive organs (Ngo et al., 2018). NPC3 and NPC4 are expressed in the young anthers, particularly in pollen sac tissues (Wimalasekera et al., 2010). According to the Genevestigator database, non-specific PLs (NPCs) are ubiquitously expressed in *Arabidopsis* and show high levels of expression in embryos (NPC2 and NPC3) and in the xylem (NPC3). They are also expressed during seed (NPC1 and NPC3) and rosette development (NPC3 and NPC6; Figure 1). Subcellularly, they localize mainly to membranous compartments (Figure 2). They have been found in the endoplasmic reticulum (NPC1; Krčková et al., 2015) and Golgi apparatus (NPC1 and NPC2; Krčková et al., 2015, 2018; Figure 2). In the leaves, NPC2 and NPC6 were recently localized in the plastids (Ngo et al., 2018). NPC4 and NPC5 are commonly localized in the microsomal fraction, and NPC5 in the cytosol, while NPC4 also associates with the plasma membrane (Figure 2; Nakamura et al., 2005; Gaude et al., 2008; Peters et al., 2014).

Experimental data show that crosstalk between NPCs and plant hormones may control gametophyte development and root growth and affect tissue mechanical strength. Consistently with expression patterns, NPC2 and NPC6 (hydrolyzing PC and PE) are redundantly required for male and female gametophyte development in *Arabidopsis* (Ngo et al., 2018). Gametophyte development also requires the expression of the PI-dependent PLC2, suggesting that some PLs may act together in this process (Figure 1). Other NPCs, such as NPC3 and NPC4, are inducible by auxin and control primary root growth and lateral root initiation under brassinolide regulation (Figure 3B; Wimalasekera et al., 2010) and root hair growth during Pi starvation (Su et al., 2018). In addition, NPC4 positively modulates ABA responses during seed germination and root elongation (Peters et al., 2010), while NPC5 positively regulates lateral root formation, perhaps by promoting auxin signaling (Peters et al., 2014).

According to expression studies based on the technique using the promoter-ß-glucuronidase (GUS), PI-PLCs, including PLC2, PLC3, and PLC5, are expressed throughout the whole plant but are mainly restricted to the vasculature. PLC2 and PLC5, unlike PLC3, show increased expression in the root tips, but all three PLCs are expressed at high levels in the reproductive organs (Kanehara et al., 2015; Zhang et al., 2018a,b). Specifically, PLC2 shows tissue-specific expression in the embryo sac during female gametophyte development, including in the maternal tissues of the ovule at the chalazal pole and in the embryo dermatogen at the late globular stage (di Fino et al., 2017).

In accordance with the abovementioned experimental results, Genevestigator data confirmed the high expression levels of PI-PLCs in the pollen (PLC4, PLC5, PLC7, and PLC8), embryos (PLC7), and phloem (PLC3). PLC2, which is expressed at the highest levels among the PLCs, shows elevated expression together with that of PLC1 during rosette development and seed formation (Figure 1). In agreement with this finding, PLC2 represents a major PL catalyzing PIP2 hydrolysis (Kanehara et al., 2015). PI-PLCs (PLC2, PLC3, and PLC9) localize to the PM (Zheng et al., 2012; Kanehara et al., 2015; di Fino et al., 2017; Ren et al., 2017), while PLC3 is localized in the nucleus (Figure 2; Ren et al., 2017).

Genetic depletion of PI-PLCs leads, in some cases, to generally reduced plant growth and a dwarf phenotype (Table S1). This is obvious for PLC2 (Kanehara et al., 2015; Chen et al., 2018) and PLC5 (Zhang et al., 2018b), while deficiencies in PLC3 (Zhang et al., 2018a) and PLC4 (Xia et al., 2017) show either very slight growth retardation or a wild type-like phenotype. *Arabidopsis* PLC2 is implicated in auxin biosynthesis, which controls the development of male and female gametophytes (Figure 3A; Li et al., 2015, di Fino et al., 2017) and roots and shoots (Figure 1; Kanehara et al., 2015, Chen et al., 2018), as well as the early stages of embryogenesis (di Fino et al., 2017). In addition, PLC2 influences also the polar distribution of auxin efflux carrier PIN2, which thus affects root hair formation and gravitropism (Figure 3D; Chen et al., 2018). These roles are likely linked to the known roles of phosphoinositides in membrane transport (Noack and Jaillais, 2017). The mode of action of *Arabidopsis* PLC3 is different from that of PLC2, and *plc3* knock-down mutants show no defects in their fertility and generative organ development. PLC3 controls lateral root development and seed germination, and it promotes ABA signaling (Zhang et al., 2018a). In addition, PLC3 functions redundantly with PLC9 in *Arabidopsis* thermotolerance (Gao et al., 2014). Importantly, PLCs (due to their phosphoinositide-hydrolyzing and PA-producing activity) control developmental processes that depend on membrane transport, such as the polar tip growth of root hairs and pollen tubes (Dowd et al., 2006; Helling et al., 2006; Pleskot et al., 2012; Hong et al., 2016). Some authors have suggested that they are also associated with the actin cytoskeleton (Huang and Crain, 2009; Andreeva et al., 2010) and thus serve as proteins connecting the actin cytoskeleton and membrane transport. Finally, pharmacological experiments revealed that PLCs and PLDs are determinants of asymmetrical cell divisions but not the cell division plane orientation, during stomatal development in maize (Apostolakos et al., 2008).

## Cellular and Developmental Roles of Phospholipases D

PLDs catalyze the hydrolysis of structural glycerophospholipids (e.g., PC, PE, and phosphatidylglycerol), producing PA and free soluble head groups (e.g., choline or ethanolamine) (Selvy et al., 2011; Hong et al., 2016). Plant PLDs can be subdivided into six classes: α (α1, α2, and α3), β (β1 and β2), γ (γ1, γ2, and γ3), δ, ε, and ζ (ζ1 and ζ2) (Hong et al., 2016). According to published data, they are mostly localized to the PM (PLDγ, PLDε, and PLDδ; Figure 2; Fan et al., 1999; Hong et al., 2009; Pinosa et al., 2013), tonoplast (PLDζ2; Su et al., 2018), nucleus and mitochondria (PLDγ; Fan et al., 1999), and cytoplasm (PLDα1; Novák et al., 2018; Figure 2). PLDα1 is capable of relocalization to the PM under conditions of salt stress (Figure 2; Novák et al., 2018). PLDζ2 is localized to the tonoplast, and upon phosphate deprivation, it accumulates in domains in the tonoplast preferentially positioned close to the mitochondria and chloroplasts (Su et al., 2018).

The expression patterns of PLDs are developmentally controlled (Fan et al., 1999; Novák et al., 2018) and tissue-dependent (Li and Xue, 2007; Hong et al., 2008; Yamaguchi et al., 2009).

Since PA is a direct product of the phospholipid-hydrolyzing activity of PLDs, many developmental and stress-related roles may be assigned to PA production. PA binds to a diverse array of substrates, including kinases (e.g., mitogen-activated protein kinase 6, MPK6; Yu et al., 2010), enzymes (e.g., glyceraldehyde 3-phosphate dehydrogenase; Kim et al., 2013), and proteins regulating the cytoskeleton (e.g., microtubule-associated protein 65–1, MAP65–1; Zhang et al., 2012) and signaling (e.g., 14–3-3 proteins; Camoni et al., 2012). PA can recruit regulatory proteins to membranes and affect diverse developmental processes (Gao et al., 2013). In addition, PLDs bind and modulate the functions of important signaling and structural proteins, such as components of the G-protein complex (Mishra et al., 2006; Roy Choudhury and Pandey, 2016), sphingosine kinase (Guo et al., 2012), MPK3 (Vadovič et al., 2019), and PLD-regulated protein1 (Ufer et al., 2017). Single T-DNA insertional mutants of PLD isoforms (PLDα and PLDδ) exhibited reduced production of PA (Hong et al., 2008), while a more drastic reduction in PA production was observed by carrying out the simultaneous mutation of both of these PLD isoforms (Bargmann et al., 2009; Uraji et al., 2012). These reports suggest that the total PA pool is generated by the additive actions of several PLDs, as well as by PI-specific PLCs. Interestingly, the reduction in PA production was much more pronounced under stress, which likely explains why *Arabidopsis* with mutant PLDs possess rather conditional phenotypes (Table S1; Li et al., 2006a; Zhao et al., 2011; Pandey, 2016).

Several of the developmental roles of PLDs depend on stress and hormonal stimuli (Table S1) because PLDs are activated in response to stress factors and hormones, which are accompanied by PA accumulation (Lanteri et al., 2008; Figure 3C). PLDα1 is important for the ABA-mediated regulation of seed germination, which depends on G-protein signaling (Roy Choudhury and Pandey, 2016). A direct physical interaction was recently reported between MPK3 and PLDα1, showing that their genetic interaction hinders the ABA-dependent inhibition of seed germination (Vadovič et al., 2019). PLDs and PA control the distribution of auxin by at least two different mechanisms. In the first of these, PLDζ2-generated PA interacts with and recruits the scaffolding of the A1 subunit of protein phosphatase 2A to the membrane, which is followed by the dephosphorylation of the auxin efflux carrier PIN1 in *Arabidopsis* (Gao et al., 2013). The second mechanism is connected to the well-known ability of PLDs to regulate membrane transport (described below). PLDζ2 is required for the recycling of PIN2-containing vesicles, as well as for polar auxin transport and distribution (Figure 3D; Li and Xue, 2007).

PLDs are regularly involved in membrane transport, which together with the activity of the cytoskeleton is a major process required for plant development. PLDα1 is required for the homeostasis of proteins involved in vesicular trafficking, membrane fusions, and ER-PM contact sites (Takáč et al., 2019b). PLD-generated PA can regulate membrane transport by causing the direct modification of membrane curvature or by recruiting important regulatory proteins (Donaldson, 2009). These proteins positively affect protein internalization (Li and Xue, 2007; Antonescu et al., 2010) and vesicle fusion (Roth, 2008). PLDα1 was also shown to interact with the adaptor-protein 2 complex, which controls the uptake and sorting of the proteins internalized by clathrin-mediated endocytosis (Yamaoka et al., 2013). This is consistent with the enhanced accumulation of PLDα1-YFP in the cortical cytoplasmic layer of *Arabidopsis* cells (Novák et al., 2018). Interestingly, the ARC1 E3 ubiquitin ligase-dependent ubiquitination of *Brassica napus* PLDα1 leads to the inhibition of multivesicular body (MVB) exocytosis in stigmas, resulting in self-incompatibility (Scandola and Samuel, 2019).

Further, PLDs and PA may regulate the nuclear localization of transcription factors important for stress responses or plant development (Yao et al., 2013; Janda et al., 2015). After the binding of the PA generated by PLDζ1 to the WER (WEREWOLF) protein, WER is translocated to the nucleus, which hinders root hair formation and elongation (Yao et al., 2013). PLDζ2 controls root and root hair elongation under phosphate-limited conditions. The exact mechanism is not known, although lipid remodeling is thought to have an important role in this regulation (Su et al., 2018).

A recent proteomic study suggested there is an integrative role of PLDα1 in chloroplast protein import and processing, as well as in plastid and cytosolic translation during chloroplast biogenesis in *Arabidopsis* (Takáč et al., 2019a). Nevertheless, the molecular mechanism linking PLDα1, which is absent in the chloroplasts, to translation and chloroplast biogenesis remains unknown. One possible link could arise from the ability of ABA to modulate the scaffold protein RACK1, which can regulate translation (Guo et al., 2011) and interact with PLDα1 (our unpublished results).

PLDs are also important regulators of cytoskeletal organization in plant cells (Pleskot et al., 2013). PLDβ1, PLDβ2, PLDγ1, PLDγ2, and PLDγ3 contain an actin-binding domain in their amino acid sequences (Hong et al., 2016). It was previously shown experimentally that tobacco PLDβ1 interacts with actin, and its enzymatic activity is enhanced by F-actin and inhibited by G-actin (Pleskot et al., 2010). On the other hand, reductions in the levels of PLD-produced PA leads to perturbed actin organization, which is mediated by the binding of PA to an actin-capping protein (Huang, 2006). In addition to actin, PLDs also bind to microtubules (Dhonukshe et al., 2003; Zhang et al., 2017b; Angelini et al., 2018; Figure 2), and MAP65-1 was found to be a direct target of PA that is important for microtubule regulation. In this way, PLDα1-derived PA controls microtubule organization during salt stress and enhances the tolerance of *Arabidopsis* to this stress (Zhang et al., 2012). This type of regulation is strictly limited to stressful conditions because the organization of cortical and mitotic microtubules in the *pldα1* mutant does not differ from that in the wild type. We recently reported that PLDα1 decorates cortical microtubules and links them with clathrin (Figure 2; Novák et al., 2018), perhaps forming a putative scaffold for the molecular players involved in the endocytic machinery. Surprisingly, a tight connection of PLDα1 with cytokinetic microtubules was also revealed, especially at the trailing (inner) edge of the enlarging phragmoplast. In addition, PLDα1 was found in the preprophase band and mitotic spindle. Thus, PLDα1 likely contributes to vesicle trafficking events connected with the delivery of membranous material to the newly forming cell plate (Novák et al., 2018). However, the possibility cannot be excluded that this PLDα1-microtubule interaction determines the activation status of PLDα1, since PLDα1 is activated by treatment with cytoskeletal inhibitors (Zhang et al., 2017a).

## Conclusions and Future Prospects

PLs have been intensively studied for more than two decades due to their ability to modulate plant stress responses. Our review points to their important roles in plant development, which are tightly interlinked with the cellular regulation and subcellular localization of PLs. Generally, PLs show overlapping functions and cooperatively control diverse developmental processes independently of their biochemical properties (Figure 1, Table S1). The developmental roles of PLs are regulated in time-, tissue- (Figure 1), and subcellular localization-dependent manners (Figure 2). The expression patterns of PLs are tightly controlled by signaling and phytohormone-dependent mechanisms. The mechanisms of PL-hormone signaling crosstalk are also diverse. PLs may regulate hormonal biosynthesis or promote the transcription of hormone-responsive genes. Moreover, such crosstalk affects the polar distributions of master regulators of hormonal signaling (Figure 3). In general, crosstalk between PLs and hormones seems to be essential to plant growth and development.

Nevertheless, little is known about the molecular regulation of the expression and activity of PLs. For example, NPC4 might be controlled transcriptionally by the homeodomain protein ALFIN-LIKE6 (Chandrika et al., 2013). However, its expression might also be controlled by post-translational modifications, such as phosphorylation (Rietz et al., 2010; Umezawa et al., 2013) or ubiquitination (Scandola and Samuel, 2019). Finally, the tissue- or cell-specific expression patterns of PLs, along with the proper subcellular localization and function of PLs and their enzymatic products, appear to control diverse developmental processes in plants. Therefore, in the future, efforts should be devoted to the more detailed elucidation of spatiotemporal patterns in the expression and subcellular localization of PLs during plant development. Recently, major advances were achieved in the determination of the developmental and subcellular localization of PLDα1 using advanced light sheet and super-resolution microscopy (Novák et al., 2018). In conclusion, more systematic studies have to be conducted to clarify the molecular mechanisms that control the expression, subcellular localization, and developmental functions of PLs.

## Author Contributions

All authors listed have made a substantial, direct and intellectual contribution to the work, and approved it for publication.

### Conflict of Interest Statement

The authors declare that the research was conducted in the absence of any commercial or financial relationships that could be construed as a potential conflict of interest.

## Supplementary Material

The Supplementary Material for this article can be found online at: https://www.frontiersin.org/articles/10.3389/fpls.2019.00362/full#supplementary-material
