# Supplementary material for: Recent Advances in the Cellular and Developmental Biology of Phospholipases in Plants
Source: Front Plant Sci. 2019 Apr 5;10:362. doi: 10.3389/fpls.2019.00362 (PMC6459882; doi:10.3389/fpls.2019.00362)
Supplement: Supplementary file 1 [file Data_Sheet_1.PDF]

Supplementary Material

Recent advances in cell and developmental biology of phospholipases in plants

Tomáš Takáč, Dominik Novák, Jozef Šamaj*

*** Correspondence:** Jozef Šamaj, jozef.samaj@upol.cz

Table S1. Overview of Arabidopsis phospholipase mutants with described phenotypes.

PLs	Gene accession number	Accession	Other name	Ecotype	Mutation	Insertion position	Phenotypes	Conditional phenotypes	References
PA-PLA$_1$	At1g31480		*sgr2-1, -2, -3, -4, -5, -6*	Col-0	EMS	Exon 19, 4, 11, 19, 8, 8	impaired shoot gravitropism, seedlings with three cotyledons		(Kato et al., 2002)
PLA$_1$Iα1	At1g05800		*pla-Iα1 7-1*	Col-0	RNAi		WT like		(Ellinger et al., 2010)
	At1g05800		*pla-Iα1 8-1*	Col-0	RNAi		WT like		(Ellinger et al., 2010)
PLA$_1$Iβ1	At2g44810		*dad1*	Ws	T-DNA, pBIH1-IG		defects in anther dehiscence, pollen maturation, and flower opening		(Ishiguro et al., 2001)
PLA$_1$Iβ2	At4g16820	SM_3_20786	*pla-Iβ2*	Col-0	T-DNA		WT like		(Ellinger et al., 2010)
PLA$_1$Iγ1	At1g06800	RATM12_1251_1	*pla-Iγ1*	No-0	T-DNA		WT like		(Ellinger et al., 2010)
PLA$_1$Iγ2	At2g30550	SALK_003105	*pla-Iγ2*	Col-0	T-DNA		WT like		(Ellinger et al., 2010)
PLA$_1$Iγ3	At1g51440	SALK_004710	*pla-Iγ3*	Col-0	T-DNA		WT like		(Ellinger et al., 2010)
PLA$_1$IIγ	At4g18550	SM_3_26312	*dsel-1*	Col-0	T-DNA	Exon 4	better root growth		(Kim et al., 2011)
	At4g18550	SGT_3532	*dsel-2*	Ler-0	T-DNA	Exon 3	better root growth		(Kim et al., 2011)
PLA$_1$III	At1g30370	WiscDsLox489-492N	*dlah*	Col-0	T-DNA	upstream Exon 1	shorter roots		(Seo et al., 2011)Seo et al., 2011
PLA$_2$β	At2g19690		*pla2β*	Col-0	RNAi		impaired cell elongation, short leaf petioles and stems		(Lee, 2003)
pPLAI	At1g61850		*plaI-1*	Ws	T-DNA	Exon 2	n.a.	reduced number of new leaves after *Botrytis cinerea* infection	(Yang et al., 2007)
			plaI-2	Ws	T-DNA	Exon 16	n.a.	reduced number of new leaves after *Botrytis cinerea* infection	(Yang et al., 2007)
		SALK_061667	*ppla-I-3*	Col-0	T-DNA	Exon 2	delayed flowering		(Effendi et al., 2014)
pPLAIIβ	At4g37050		*plaIVc-1*	Ws	T-DNA	Exon 6	WT-like		(Rietz et al., 2010)
pPLAIIγ	At4g37070	SALK_036785.54.90.x	*plaIVa-1*	Col-0	T-DNA	Intron 2	altered lateral root development		(Rietz et al., 2010)
		SALK_027625.39.00.x	*plaIVa-2*	Col-0	T-DNA	Intron 2	altered lateral root development		(Rietz et al., 2010)
pPLAIIδ	At4g37060	SALK_073180.18.00.x	*plaIVb-1*	Col-0	T-DNA	Exon 4	WT-like		(Rietz et al., 2010)
		SALK_090933.54.20.x	*plaIVb-2*	Col-0	T-DNA	Exon 7	WT-like		(Rietz et al., 2010)
pPLAIIIβ	At3g54950	SALK_057212	*ppla-IIIβ*	Col-0	T-DNA	Exon 1	longer leaves, petioles, hypocotyls, primary roots and root hairs		(Li et al., 2011)
pPLAIIIδ	At3g63200		*sturdy*	Col-0	dominant GOF	Exon 2	stiff inflorescence stems, thicker leaves, shorter siliques, larger seeds, round-shaped flowers and		(Huang, 2001)

					mutant		delayed growth		
		SALK_029470	*ppla-IIIδ*	Col-0	T-DNA			hypersensitivity to auxin with decreased primary root length and increased lateral root number	(Labusch et al., 2013)
PI-PLC2	At3g08510	SALK_152284	*plc2*	Col-0	T-DNA	Exon 8	altered reproductive organ development		(Li et al., 2015; Di Fino et al., 2017)
		FLAG_506C04	*plc2-1*	Ws (Col-0)		Intron 1	growth retardation, reduced root length		(Kanehara et al., 2015; Di Fino et al., 2017)
			plc2-CRISPR	Col-0	CRISPR/ Cas9		altered reproductive organ development		(Li et al., 2015)
PI-PLC3	At4g38530	SALK_037453	*plc3-2*	Col-0	T-DNA	Exon 3	shorter primary roots, fewer lateral roots, reduced lateral root densities		(Zhang et al., 2018a)
		SALK_054406	*plc3-3*	Col-0	T-DNA	Intron 3			
PI-PLC4	At5g58700	SALK_201150	*plc4*	Col-0	T-DNA	Exon 2	WT like	hyposensitivity to salt stress, higher fresh weight and primary root length	(Xia et al., 2017)
PI-PLC5	At5g58690	SALK_144469	*plc5-1*	Col-0	T-DNA	Exon 7	shorter primary roots, fewer lateral roots, reduced lateral root densities		(Zhang et al., 2018b)
PI-PLC7	At3g55940	SALK_044778	*plc7-1*	Col-0	T-DNA	Intron 6	WT like		(Zheng et al., 2012)
		SALK_030333	*plc7-2*	Col-0	T-DNA	Exon 7	WT like		(Zheng et al., 2012)
PI-PLC8	At3g47290	SALK_150154	*plc8-1*	Col-0	T-DNA	Intron 7	WT like		(Zheng et al., 2012; Xia et al., 2017)
PI-PLC9	At3g47220	SALK_120782	*plc9-1*	Col-0	T-DNA	Exon 4	WT like	delayed growth after heat stress	(Zheng et al., 2012)
		SALK_025949	*plc9-2*	Col-0	T-DNA	Intron 2	WT like	delayed growth after heat stress	(Zheng et al., 2012; Xia et al., 2017)
NPC1	At1g07230	SAIL_548H09	*npc1-2*	Col-0	T-DNA	Exon 1	WT like		(Ngo et al., 2018)
NPC2	At2g26870	SALK_018011	*npc2-1*	Col-0	T-DNA	Intron 1	WT like		(Ngo et al., 2018)
NPC3	At3g03520	SALK_036463.4 6.20.x	*npc3-1*	Col-0	T-DNA		slightly reduced primary root length	reduced lenght of primary roots, increased length and density of lateral root after phophate starvation, auxin and brassinolide-mediated reduction of primary root elongation and lateral root density.	(Wimalasekera et al., 2010)
		SALK_150666.2 3.25.n	*npc3-2*	Col-0	T-DNA				
NPC4	At3g03530	SALK_046713.4 7.10.x	*npc4-1*	Col-0	T-DNA		slightly reduced primary root length	reduced primary roots, increased length and density of lateral root after phophate starvation, auxin and brassinolide-mediated reduction of primary root elongation and lateral root density.	(Wimalasekera et al., 2010)
		GK571E10	*npc4-2*	Col-0	T-DNA				
NPC5	At3g03540	SALK_045037	*npc5-1*	Col-0	T-DNA	Exon 3	WT like	few to no lateral roots under mild NaCl stress	(Peters et al., 2014)
NPC6	At3g48610	SALK_048020	*npc6-2*	Col-0	T-DNA	Exon 2	WT like		(Ngo et al., 2018)
PLDα1	At3g15730	SALK_067533	*pldα1-1*	Col-0	T-DNA	Exon 3	WT like		(Bargmann et al., 2009; Pandey, 2016)
		SALK_053785	*pldα1-2*	Col-0	T-DNA	Exon 2	WT like		(Bargmann et al., 2009; Zhang et al., 2004)
PLDα3	At5g25370	SALK_130690	*pldα3-1*	Col-0	T-DNA	Exon 2	WT like	delayed germinatiom, smaller seedling size, shorter primary roots, fewer lateral roots under salt stress, delayed flowering after drought stress.	(Hong et al., 2008)
PLDγ1	At4g11850	SALK_113873	*pldγ1-1*	Col-0	T-DNA	Exon 8	WT like	better root growth under Al stress	(Zhao et al., 2011)

PLDγ2	At4g11830	SALK_014510	*pldγ2-1*	Col-0	T-DNA	Intron 6	WT like		(Zhao et al., 2011)
PLDε	At1g55180	SALK_023603	*pldε*	Col-0	T-DNA	Exon 2	WT like	reduced root growth and biomass accumulation during hyperosmotic stress	(Hong et al., 2009)
PLDζ1	At3g16785	SALK_083090	*pldζ1*	Col-0	T-DNA	Exon 1	WT like		(Li et al., 2006)
PLDζ2	At3g05630	SALK_094369	*pldζ2*	Col-0	T-DNA	Exon 9	WT like		(Li et al., 2006)

Supplemental references

Bargmann, B. O. R., Laxalt, A. M., Riet, B. t., van Schooten, B., Merquiol, E., Testerink, C., et al. (2009). Multiple PLDs Required for High Salinity and Water Deficit Tolerance in Plants. *Plant Cell Physiol.* 50, 78–89. doi:10.1093/pcp/pcn173.

Di Fino, L. M., D'Ambrosio, J. M., Tejos, R., van Wijk, R., Lamattina, L., Munnik, T., et al. (2017). Arabidopsis phosphatidylinositol-phospholipase C2 (PLC2) is required for female gametogenesis and embryo development. *Planta* 245, 717–728. doi:10.1007/s00425-016-2634-z.

Effendi, Y., Radatz, K., Labusch, C., Rietz, S., Wimalasekera, R., Helizon, H., et al. (2014). Mutants of phospholipase A (pPLA-I) have a red light and auxin phenotype: Phospholipase A pPLA-I in auxin and light signalling. *Plant Cell Environ.* 37, 1626–1640. doi:10.1111/pce.12278.

Ellinger, D., Stingl, N., Kubigsteltig, I. I., Bals, T., Juenger, M., Pollmann, S., et al. (2010). DONGLE and DEFECTIVE IN ANTHER DEHISCENCE1 lipases are not essential for wound- and pathogen-induced jasmonate biosynthesis: redundant lipases contribute to jasmonate formation. *Plant Physiol.* 153, 114–127. doi:10.1104/pp.110.155093.

Hong, Y., Devaiah, S. P., Bahn, S. C., Thamasandra, B. N., Li, M., Welti, R., et al. (2009). Phospholipase Dε and phosphatidic acid enhance Arabidopsis nitrogen signaling and growth. *Plant J.* 58, 376–387. doi:10.1111/j.1365-313X.2009.03788.x.

Hong, Y., Pan, X., Welti, R., and Wang, X. (2008). Phospholipase D 3 Is Involved in the Hyperosmotic Response in Arabidopsis. *Plant Cell* 20, 803–816. doi:10.1105/tpc.107.056390.

Huang, S. (2001). Cloning of an Arabidopsis Patatin-Like Gene, STURDY, by Activation T-DNA Tagging. *Plant Physiol.* 125, 573–584. doi:10.1104/pp.125.2.573.

Ishiguro, S., Kawai-Oda, A., Ueda, J., Nishida, I., and Okada, K. (2001). The DEFECTIVE IN ANTHER DEHISCIENCE gene encodes a novel phospholipase A1 catalyzing the initial step of jasmonic acid biosynthesis, which synchronizes pollen maturation, anther dehiscence, and flower opening in Arabidopsis. *Plant Cell* 13, 2191–2209.

Kanehara, K., Yu, C.-Y., Cho, Y., Cheong, W.-F., Torta, F., Shui, G., et al. (2015). Arabidopsis AtPLC2 Is a Primary Phosphoinositide-Specific Phospholipase C in Phosphoinositide Metabolism and the Endoplasmic Reticulum Stress Response. *PLOS Genet.* 11, e1005511. doi:10.1371/journal.pgen.1005511.

Kato, T., Morita, M. T., Fukaki, H., Yamauchi, Y., Uehara, M., Niihama, M., et al. (2002). SGR2, a phospholipase-like protein, and ZIG/SGR4, a SNARE, are involved in the shoot gravitropism of Arabidopsis. *Plant Cell* 14, 33–46.

Kim, E. Y., Seo, Y. S., and Kim, W. T. (2011). AtDSEL, an Arabidopsis cytosolic DAD1-like acylhydrolase, is involved in negative regulation of storage oil mobilization during seedling establishment. *J. Plant Physiol.* 168, 1705–1709. doi:10.1016/j.jplph.2011.03.004.

Labusch, C., Shishova, M., Effendi, Y., Li, M., Wang, X., and Scherer, G. F. E. (2013). Patterns and timing in expression of early auxin-induced genes imply involvement of phospholipases A (pPLAs) in the regulation of auxin responses. *Mol. Plant* 6, 1473–1486. doi:10.1093/mp/sst053.

Lee, H. Y. (2003). Secretory Low Molecular Weight Phospholipase A2 Plays Important Roles in Cell Elongation and Shoot Gravitropism in Arabidopsis. *Plant Cell* 15, 1990–2002. doi:10.1105/tpc.014423.

Li, L., He, Y., Wang, Y., Zhao, S., Chen, X., Ye, T., et al. (2015). Arabidopsis PLC2 is involved in auxin-modulated reproductive development. *Plant J.* 84, 504–515. doi:10.1111/tpj.13016.

Li, M., Bahn, S. C., Guo, L., Musgrave, W., Berg, H., Welti, R., et al. (2011). Patatin-Related Phospholipase pPLAIII -Induced Changes in Lipid Metabolism Alter Cellulose Content and Cell Elongation in Arabidopsis. *Plant Cell* 23, 1107–1123. doi:10.1105/tpc.110.081240.

Li, M., Qin, C., Welti, R., and Wang, X. (2006). Double knockouts of phospholipases Dzeta1 and Dzeta2 in Arabidopsis affect root elongation during phosphate-limited growth but do not affect root hair patterning. *Plant Physiol.* 140, 761–770. doi:10.1104/pp.105.070995.

Ngo, A. H., Lin, Y.-C., Liu, Y., Gutbrod, K., Peisker, H., Dörmann, P., et al. (2018). A pair of nonspecific phospholipases C, NPC2 and NPC6, are involved in gametophyte development and glycerolipid metabolism in *Arabidopsis*. *New Phytol.* 219, 163–175. doi:10.1111/nph.15147.

Pandey, S. (2016). Phospholipases as GTPase activity accelerating proteins (GAPs) in plants. *Plant Signal. Behav.* 11, e1176821. doi:10.1080/15592324.2016.1176821.

Peters, C., Kim, S.-C., Devaiah, S., Li, M., and Wang, X. (2014). Non-specific phospholipase C5 and diacylglycerol promote lateral root development under mild salt stress in Arabidopsis: NPC5 and DAG promote lateral root development. *Plant Cell Environ.* 37, 2002–2013. doi:10.1111/pce.12334.

Rietz, S., Dermendjiev, G., Oppermann, E., Tafesse, F. G., Effendi, Y., Holk, A., et al. (2010). Roles of Arabidopsis Patatin-Related Phospholipases A in Root Development Are Related to Auxin Responses and Phosphate Deficiency. *Mol. Plant* 3, 524–538. doi:10.1093/mp/ssp109.

Seo, Y. S., Kim, E. Y., and Kim, W. T. (2011). The Arabidopsis sn-1-specific mitochondrial acylhydrolase AtDLAH is positively correlated with seed viability. *J. Exp. Bot.* 62, 5683–5698. doi:10.1093/jxb/err250.

Wimalasekera, R., Pejchar, P., Holk, A., Martinec, J., and Scherer, G. F. E. (2010). Plant Phosphatidylcholine-Hydrolyzing Phospholipases C NPC3 and NPC4 with Roles in Root Development and Brassinolide Signaling in Arabidopsis thaliana. *Mol. Plant* 3, 610–625. doi:10.1093/mp/ssq005.

Xia, K., Wang, B., Zhang, J., Li, Y., Yang, H., and Ren, D. (2017). *Arabidopsis* phosphoinositide-specific phospholipase C 4 negatively regulates seedling salt tolerance: AtPLC4 negatively regulates salt tolerance. *Plant Cell Environ.* 40, 1317–1331. doi:10.1111/pce.12918.

Yang, W., Devaiah, S. P., Pan, X., Isaac, G., Welti, R., and Wang, X. (2007). AtPLAI is an acyl hydrolase involved in basal jasmonic acid production and Arabidopsis resistance to Botrytis cinerea. *J. Biol. Chem.* 282, 18116–18128. doi:10.1074/jbc.M700405200.

Zhang, Q., van Wijk, R., Shahbaz, M., Roels, W., Schooten, B. van, Vermeer, J. E. M., et al. (2018a). Arabidopsis Phospholipase C3 is Involved in Lateral Root Initiation and ABA Responses in Seed Germination and Stomatal Closure. *Plant Cell Physiol.* 59, 469–486. doi:10.1093/pcp/pcx194.

Zhang, Q., van Wijk, R., Zarza, X., Shahbaz, M., van Hooren, M., Guardia, A., et al. (2018b). Knock-Down of Arabidopsis PLC5 Reduces Primary Root Growth and Secondary Root Formation While Overexpression Improves Drought Tolerance and Causes Stunted Root Hair Growth. *Plant Cell Physiol.* 59, 2004–2019. doi:10.1093/pcp/pcy120.

Zhang, W., Qin, C., Zhao, J., and Wang, X. (2004). Phospholipase D alpha 1-derived phosphatidic acid interacts with ABI1 phosphatase 2C and regulates abscisic acid signaling. *Proc. Natl. Acad. Sci. U. S. A.* 101, 9508–9513. doi:10.1073/pnas.0402112101.

Zhao, J., Wang, C., Bedair, M., Welti, R., W. Sumner, L., Baxter, I., et al. (2011). Suppression of Phospholipase Dγs Confers Increased Aluminum Resistance in Arabidopsis thaliana. *PLoS ONE* 6, e28086. doi:10.1371/journal.pone.0028086.

Zheng, S.-Z., Liu, Y.-L., Li, B., Shang, Z.- lin, Zhou, R.-G., and Sun, D.-Y. (2012). Phosphoinositide-specific phospholipase C9 is involved in the thermotolerance of Arabidopsis: AtPLC9 plays a role in thermotolerance. *Plant J.* 69, 689–700. doi:10.1111/j.1365-313X.2011.04823.x.
